# Supplementary material for: The Rapid Adaptation and Optimisation of a Digital Behaviour-Change Intervention to Reduce the Spread of COVID-19 in Schools
Source: Int J Environ Res Public Health. 2022 May 31;19(11):6731. doi: 10.3390/ijerph19116731 (PMC9180389; doi:10.3390/ijerph19116731)
Supplement: Supplementary file 1 [file ijerph-19-06731-s001.zip › ijerph-1650596-supplementary.pdf]

A: PPI Topic Guide

Questions for school staff:

- What are their school's current practices around infection control? How do they involve pupils in this (if at all)?
- From your point of view/ in your role – what are the most challenging aspects of managing Covid-19 in schools?
- Which other school staff are involved in helping manage COVID?
- What (if anything) is missing from the current COVID-security policies?
- How do the messages in Germ Defence fit (or not) with current practice?
- Are the messages in Germ Defence useful?
- Would they use Germ Defence to inform infection control in their homes / at school? Why/Why not?
- Any content missing that they feel is important to add?
- How would they envisage using with/disseminating to pupils in practice (if at all)?
- We'd like to get your thoughts on each of the specific behaviours that Germ defence will focus on: Handwashing, mask wearing, social distancing and keeping the space around you clean (and well ventilated).
- We would also like to get your thoughts about how we're proposing to change/ restructure Germ Defence and what some of the key content is that we are proposing.

Questions for students:

- How well do you feel that you know/ understand the guidelines – in school? Outside of school? How different are they? How about your friends – how much do they know/understand them?
- How important do you think it is to follow the rules, even if you're not considered to be at risk yourself? Why/ Why not?
- Would you be more likely to follow the rules for yourself or others? Which others? What about people you don't know?
- How important do you think the information, messages and ideas in Germ Defence are? Why?
- Would you use it? Would you friends use it? Why/ why not?
- When/ where would they use it? On phones/computers? At school/home?

- What would they change about it?
- What do they think of the behaviours that it is advising? E.g., handwashing

Questions for parents:

- What are current practices (if known) around infection control practices at their child's school?
- What are the most challenging aspects of managing covid-19 in schools for yourself? For your child?
- What do they think of the key messages in Germ Defence (e.g., risk messages / greater good / small things can have a big impact)
- What do you think about the behaviours that it is advising? E.g., handwashing, distancing, mask wearing.
- How easy (or otherwise) would it be to implement the behaviours? Themselves? Their children? In schools?
- Are there any infection control behaviours (school or home) that are missing?
- Do they think Germ Defence could be used in their child's school?
- If their child's school was using it, and inviting children and parents to use it, how would they feel? Would they have any concerns?
- What would they change about it?

B: Intervention Planning Table

Key Features	Evidence and Source
**Guiding principle 1: Minimising work required to engage with Germ Defence**	
• Simple, short pages with minimal text<br>• Optimise content and structure for mobile access<br>• Simple navigation and consistent page structure<br>• Separation of home and school sections to facilitate rapid identification of relevant information<br>• Align 'home' and 'school' sections to same modular design e.g., Handwashing, Social distancing, Wearing face coverings & Keeping your surroundings safe<br>• Accompanying 'How to Use' guide for school staff	**School staff & student PPI discussions:**<br>• Students preferred shorter content, and they would be more likely to engage if intervention was also sent home.<br>• Need to separate guidelines for home and school as rules differ within each.<br>• An accompanying document to provide staff with ideas about how to use Germ Defence was welcomed.<br><br>**Evidence from literature:**<br>• Staff have extensive, additional responsibilities as a result of COVID-19 measures [6].<br>• Staff felt overwhelmed by the ever-changing, unclear guidance from Government, the amount of legislation and school policies they were required to read and implement [6].<br>• Future interventions for school-based settings should target multifaceted approaches i.e., focussing on a range of environmental disinfecting behaviours, including cleaning surfaces, rather than handwashing alone [7].<br>• Such approaches are likely to be most successful when combined with 'soft' hygiene promotion to address a range of determinants rather than only providing education regarding disease transmission [21].
**Guiding principle 2: Promoting collective responsibility**	
• Frame risk messages in terms of protecting vulnerable others and looking after whole community<br>• Use of social norms to encourage (especially young people) to adhere recommendations/not go along with peers who may not be<br>• Highlight benefits of promoted actions for self AND others	**School staff, parent PPI discussions**:<br>• Staff reported that young people wish to return to normal as soon as possible and will find excuses not to wear face coverings or wash their hands regularly.<br><br>**Evidence from literature:**<br>• A lack of understanding by young people of collective need is a potential barrier [6].

	• Staff report emphasising the 'collective good' may increase compliance with risk reduction measures amongst students [6].
**Guiding principle 3: Facilitate understanding of risk reduction vs. elimination objective**	
• Acknowledge difficulties in implementing all measures all the time and the need for adaptation to user context.<br>• Staged/stepped behavioural suggestions – starting with optimal solution and then alternatives if this not possible<br>• Acknowledge users' current behaviours and provide persuasive suggestions as to how these can change.<br>• Provide encouragement for positive changes to planned, future behaviours.	**School staff, parent and student PPI discussions:**<br>• Staff were sceptical of the feasibility of planned measures.<br>• Staff felt important messages about risk reduction vs elimination and viral load had not previously been made clear enough.<br>• Staff understood that following suggested behaviours did not need to be perfect but was still worth doing.<br>• Students felt measures could be less stringent as key messages at the time from media/scientists stated young people were less likely to contract Covid-19 or become ill from it, leading some young people to have a relaxed attitude towards adopting health behaviours.<br>• Difficulties were voiced by parents and students regarding 'following the rules' when other people were not doing so.<br><br>**Evidence from literature:**<br>• Parents, students and staff felt distancing in schools would be difficult and risk reduction measures would impact on learning [6].<br>• Staff and parents were concerned of a lack of engagement with appropriate behaviours and staff believed students generally follow rules, but they had concerns about how to enforce them [6].
**Guiding principle 4: Facilitate understanding of reasons for each behavioural recommendation**	
• Present strong, accessible, rationale for all behavioural recommendations (including explaining why recommendations change and vary)	**School staff, parent and student PPI discussions:**<br>• Students acknowledged the importance of measures, but were sometimes confused by them i.e., why rules varied in different contexts. Also, general confusion about 'bubbles'.<br>• Staff stressed the importance of rationale for students adopting health behaviours.

• Acknowledgement that measures may sometimes seem confusing/contradictory but explain in terms of risk reduction.<br>• Suggestions for strategies for managing situations/contexts where desired behaviours difficult/ not possible<br>• Encourage habit-forming behaviours by asking users to make a plan of future recommended behaviour.<br>• Explain when it is most important to perform key behaviours (e.g., wash/sanitise hands when coming in from outside, before eating, when touching shared objects, after coughing or sneezing and after visiting the toilet).<br>• Provide a printable summary 'poster' to be displayed within the classroom/home providing reminders about key behaviours.	• Some parents highlighted inconsistencies between messages between home and school - this was confusing for students who received different messages from their friends.<br>• Parents acknowledged schools offered a good opportunity to provide consistent messages, schools had the authority to share these messages with their staff, parent and student communities.<br>• Staff need clear advice to share with students and parents regarding the importance of health behaviours.<br>• Staff advised providing specific examples of appropriate times to wash hands etc.<br><br>**Evidence from literature:**<br>• Sustained efforts are needed to ensure uptake in handwashing routine care. Interventions must not only remind children to wash their hands but also teach them how to do so effectively [18].<br>• Reminders and information regarding handwashing are not sufficient. Structural factors, including having the time to wash hands, and the facilities to do so, will also influence hand washing behaviour [19].<br>• Measures to encourage the habit of frequent handwashing are essential [20].<br>• Hygiene promotion interventions are more likely to be successful with soft, empathetic approaches to hygiene promotion which acknowledges a range of determinants [21].<br>• Handwashing interventions targeted towards children should be persuasive as well as instructional [23].<br>• Need to increase recognition of the potential for airborne spread of Covid-19 [26]; associated prevention measures, such as ventilation, must be advocated to mitigate airborne transmission of viruses [27] including Covid-19 [28].
**Guiding principle 5: Persuade users that within-home transmission is not inevitable**	

• Recommendations and strategies for implementing measures used outside the home within the home.<br>• Advice about dealing with visitors to the home and how to manage social expectations.<br>• Recommendations presented in format/structure that recognises that more stringent measures may only be feasible/acceptable in the home under certain circumstances/ in certain contexts.	**School staff, parent and student PPI discussions:**<br>• Students believed contracting Covid-19 in the home was inevitable from a positive case, but that the same did not apply in school as this was a more controlled environment.<br>• Staff felt wearing a face covering was more widely accepted when people were 'out and about' but less so when inside the home.<br>• Social distancing and wearing face coverings within the home was not perceived by parents as feasible or realistic, especially when with young children.<br>• Parents believed contracting Covid-19 in the home was inevitable from a positive case.<br><br>**Evidence from literature:**<br>• Ventilation, face coverings, handwashing and social distancing measures successfully reduce the risk of secondary infection within a household/classroom [34].<br>• Virus transmission within the home is preventable and the value of perseverance and habit-changing should be emphasised [28].

C: Think Aloud Interview Schedule

<u>Pre-Think Aloud Questions:</u>

Questions for students:

- Can you tell me a bit about who you live with?
    - Do you live with anyone who is considered vulnerable/high-risk?
- Can you tell me a bit about what you know about coronavirus?
- How serious do you think it is?
    - Why?
- What sort of things have you done to try and stay safe?
- Can you tell me about any other things that you think are important for trying to stay safe?

Thank you for sharing your thoughts. Now if we turn to looking at the Germ Defence website, I'd like to hear what you think about that.

Questions for parents / school staff:

- Can you tell me a bit about your current living situation?
    - Are you or someone you live with considered vulnerable/high-risk?
- How has your experience of the coronavirus pandemic changed between the beginning, middle and now?
- How worried about coronavirus are you?
- How are things now that you/your child(ren) are back at school?

Thank you for sharing your thoughts. Now if we turn to looking at the Germ Defence website, I'd like to hear what you think about that.

Guidance document for teachers:

Prompts:

- Is the information clear?
- Is the guidance on how to appropriately use Germ Defence clear?
- What do you like about this document?
- What do you dislike about this document?
- What would you change about this document?
- Do you think the activity-based suggestions are appropriate? If not, why?

- Do you have any suggestions of how else Germ Defence might be used in school/ how could we make it easier for teachers to use in their classrooms?

Think Aloud Questions:

- Do you think the information on vaccinations is relevant to you and/or your students/child(ren)?
    - Do you think it's appropriate to view this information on vaccinations at the beginning of Germ Defence? If not, why?
- [school/home section split] What do you think about the split between protecting yourself and others at home or in school?
- [at home main menu] Is there enough information on 'what to do next' on this page?
- [when feedback and recruitment pop-ups appear] What are your thoughts on these questions being asked at this point?
- [goal-setting questions] What do you think about answering these behavioural questions at this point of Germ Defence (i.e., is it too early/late)?
- [participants who identified as high risk/living with someone else who is] – Researcher to note whether or not participant clicks to expand the drop-downs, and ask why they did or did not do this.
- [social distancing to protect vulnerable children drop-down] Who do you think this content is aimed at and why?
- [order of the stories] What do you think about these stories? And what do you think about viewing them at this point?

Researcher Prompts:

- [on **key pages**, e.g., menus/first page etc.] What are your first impressions of this page?
- What are you thinking now?
- What made you choose that option?
- What do you think about [this behaviour/suggestion/information/idea]?
- Can you tell me a bit more about why you think that?
- [in response to an expression of like/dislike] What is it you like/don't like about that?
- That's really interesting/a good idea… Can you tell me more?

- [picking up on facial expressions/body language] I noticed that you frowned/smiled at… Can you tell me what you thought about that?

<u>Post-Think Aloud Questions:</u>

- Overall, what do you think about Germ Defence?
- Can you tell me about anything you thought was particularly good about Germ Defence?
- Can you tell me anything about Germ Defence that you were less keen on?
- [For teachers and students **<u>only</u>**] Could you briefly tell me about your experience of coronavirus testing in school.
    - Do you think we should add some information to Germ Defence about testing and why?
- Can you tell me about anything you might change?
    - How/why would you change this?
- Can you tell me about anything else that you think might be useful to you?
- Would Germ Defence be useful for use in your/your child's school?
    - How do you think it could be used?
- What would you think if you/your child's school asked you to use the website?
- What did you think of the behaviours mentioned in Germ Defence?
- Which of the behaviours mentioned in Germ Defence would you be willing to try?
    - Why these ones?
- Could you tell me about anything that would make it easier to try these [behaviours]?
- Could you tell me about anything that would make it difficult to try the behaviours suggested in the Germ Defence?
- In what ways do you think these difficulties could be resolved?
- Can you tell me about possible advantages of following the advice from the website?
- Can you tell me about possible disadvantages of following the advice from the website?
- Could you tell me about the type of support that you might find helpful in addition to the website?

D: Excerpts from Table of Changes

Section	Negative Comments[1]	Positive Comments	Possible Change	Reason for change (EASy, IMPortant, REPetitive)	Agreed change	MoScoW
Initial introduction page		I like the presentation of this page, I like the little diagrams. They're kind of the same for each little picture. So, anything colourful with children is good (T8)			No change.	
Initial introduction page	I think a lot of children do think as well, 'It's ending, people are getting vaccinated, and things are turning back to normal – whatever normal actually is,' so, again, why are they learning about COVID and how to stop the spread when things are changing?... there needs to be some kind of acknowledgement within the		Add reference to other viruses.	EAS	Add in a sentence to this page - for example, "Germ Defence is useful to help reduce the spread of coronavirus and other viruses such as the flu, norovirus and stomach bugs".	Could have

[1] Similar comments, given by more than one participant, have been paraphrased.

	website perhaps… because, even though it's getting better than what it was, we still need to all do our bit to prevent COVID and other viruses (P9)					
'Cutting down on germs' page		I didn't know that avoiding as much viruses as we can will help protect ourselves and others from becoming seriously ill, I thought it would be just us, just me (C7)			No change.	
Menu page in both Home and School section (vertical list of behaviours –'what would you like to find out about first?')	Rather than just 'Handwashing,' it should be, 'Handwashing and Sanitising', because we don't give the students the opportunity to wash their hands regularly within school – they come into the classroom, they sanitise their hands and then, when they leave the classroom, they sanitise their hands, but they don't wash them as such (T3)		Rename menu title as suggested.	EAS, IMP	Change to 'keeping your hands free of virus' (in both home and school sections, for consistency).	Could have
Vaccination and testing		Once the vaccinations [are] going to be rolled out			No change.	

information page		to the students and to young adults, that's good for them just to have that bullet point, just to know where they're at after the first vaccination and how to prepare for it (P7)				
'Dropdowns' of additional information for high-risk users	I won't click that because it looks like a hyperlink and I don't want to leave the website, get lost, and not be able to come back to it. (C4) (P7) (P2) (P4)		To change the appearance of the dropdown titles.	REP, IMP	Edit the font colour of the drop-down titles, to **not** have them underlined, **and** to insert an arrow pointing downwards if possible.	Must have
Suggested behaviours	I don't know if somehow you could add hand sanitiser, which I know it's not better than washing your hands but if you've left the house and gone in the car to Tesco you can't wash your hands before you put a face mask on (T9)		Add in 'sanitise your hands' to this sentence.	EAS, IMP	Add in 'sanitise your hands' to this sentence.	Should have
Current behaviour questions	I don't understand what the word 'applicable' means (C3) (C7) (C8) (C9) (C12)		Change point to "does not apply to me".	EAS	Change 'not applicable' to 'does not apply to me'	Could have

	Doesn't apply to me' probably would be a better way because someone [students] will ask what applicable means (T1)				throughout both home and school sections.	
Future (planned) behaviour questions	I think it would be really good if they could now click an option to view their plan. Something to print off or keep (T1) (T12)		Add a 'print plan' option.	EAS	Add a 'print plan' option.	Could have
